# Comprehensive Utilization of Thinned Unripe Fruits from Horticultural Crops

**DOI:** 10.3390/foods10092043

**Published:** 2021-08-30

**Authors:** Mengyuan Wei, Haoli Wang, Tingting Ma, Qian Ge, Yulin Fang, Xiangyu Sun

**Affiliations:** 1 College of Enology, College of Food Science and Engineering, Viti-Viniculture Engineering Technology Center of State Forestry and Grassland Administration, Shaanxi Engineering Research Center for Viti-Viniculture, Heyang Viti-viniculture Station, Northwest A&F University, Yangling 712100, China; weimengyuan@nwafu.edu.cn (M.W.); Wangholy@nwafu.edu.cn (H.W.); matingting@nwafu.edu.cn (T.M.); geqian1116@shou.com (Q.G.); fangyulin@nwsuaf.edu.cn (Y.F.); 2 Quality Standards and Testing Institute of Agricultural Technology, Yinchuan 750002, China

**Keywords:** horticultural crops, thinned unripe fruits, phenolic bioactive compounds, non-phenolic bioactive compounds, comprehensive utilization

## Abstract

Fruit thinning is a cultivation technique that is widely applied in horticulture in order to obtain high-quality horticultural crops. This practice results in the discarding of a large number of thinned unripe fruits in orchards each year, which produces a great waste of agricultural resources and causes soil pollution that may be an important reservoir for pest and plant diseases. Current studies showed that bioactive compounds such as polyphenols, organic acids, monosaccharides and starches are present in unripe fruits. Therefore, we reviewed the bioactive components obtained from thinned unripe fruits, their revalorization for the food industry, their beneficial effects for human health and the methods for obtaining these components. We also performed a calculation of the costs and benefits of obtaining these bioactive compounds, and we proposed future research directions. This review provides a reference for the effective utilization and industrial development of thinned unripe fruits obtained from horticultural crops. Furthermore, revalorizing the waste from this cultural practice may increase the economic benefits and relieve the environmental stress.

## 1. Introduction

Thinning is the removal of individual fruits after fruit set [1,2]. Many fruit species support a variable amount of fruit and have self-regulatory mechanisms for natural fruit dropping. However, trees are frequently unable to support fruit productivity using only self-regulating mechanisms [3,4,5,6,7,8]. For most horticultural crops, relying on falling off by themselves prevents an optimal crop load for the production of ripe fruits, with a balance of quality and quantity, and exogenous intervention is needed to achieve these goals. Seehuber et al. [9] reported that the nutritional composition of fruits, fruit size, yield, fruit shape and number of seeds were affected by fruit thinning. The sugar content, pH and mineral composition of fruits were also affected. To perform fruit thinning, some of the developing unripe fruits are removed to allow the remaining unripe fruits to have more photosynthetic products available to increase fruit size and improve quality [10,11,12]. Fruit thinning is also one of the best cultivation practices that may increase flower return the following season [13]. Fruit thinning balances the fruit-to-shoot ratio and increases the availability of nutrients for fruits, shoots and reserve organs [14].

**Figure 1:**
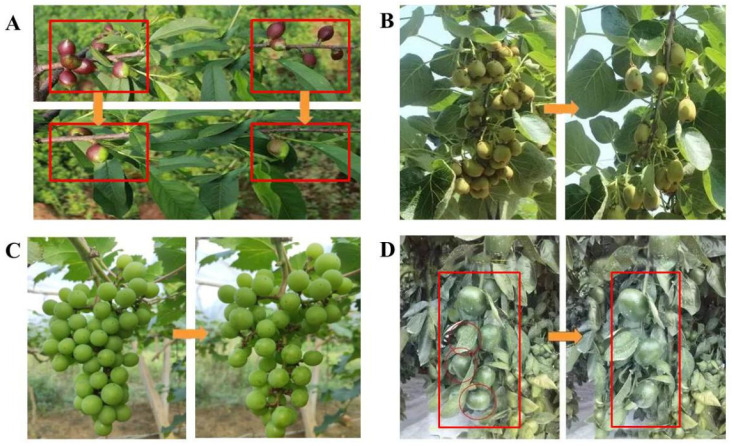
Thinning fruit: (**A**) peach, (**B**) kiwifruit, (**C**) grape, (**D**) and citrus.

Therefore, many horticultural crops, such as grapes, apples, peaches, pears, pomegranates, citruses, and cherries, need fruit thinning in their crop management strategies (Figure 1). Due to the need to improve fruit quality in crop management by physiological drop or fruit thinning, a large number of unripe fruits are discarded every year. For example, the production of grapes and apples in China generates relatively few thinned fruits. The amount of thinned fruits per acre is 30–60 kg for grapes [15], 30–50 kg for apples [16,17], nearly 100 kg for peaches and pears [15,18], and 280–400 kg for pomegranates [19].

Thinning fruit improves the economic value of horticultural crops. However, the careless handling of thinned unripe fruits will cause variable harm to the soil, plants and environment. The traditional utilization of some discarded unripe fruits could reduce the toxicity of the soil environment. Some farmers use unripe thinned fruits as poultry feed [20]. In the tropics, sheep feed is generally based on corn and soybean which must be imported from abroad. Archimède de et al. [21] substituted the imported feed with local food resources and reported that unripe discarded bananas together with local forages replaced the conventional protein supplements of corn and soybean in sheep feed. Orange waste is also effective in reducing the fat content of poultry and increasing meat content [22,23]. Additionally, discarded unripe fruits may also be used together with other agricultural wastes for composting, power generation, and fuel extraction [15,24].

**Figure 2:**
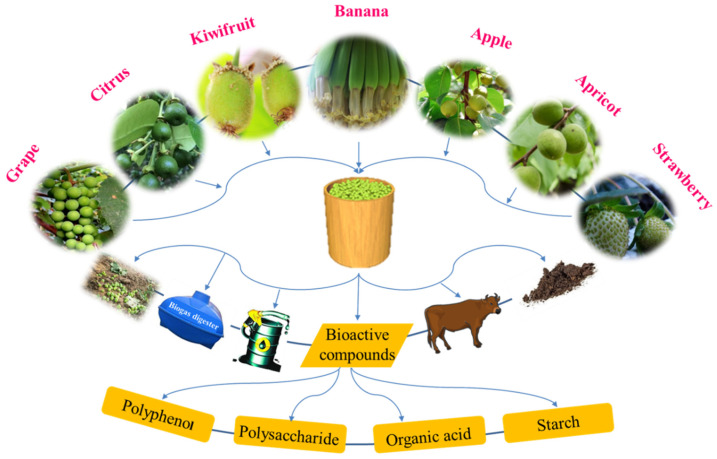
Utilization of thinned unripe fruit.

In recent years, many researchers have found bioactive compounds, such as polyphenols, organic acids, monosaccharides and starches. These are important parts of unripe fruits, and are potential beneficial resources in many industries such as human health, food preservation, functional foods, and industrial materials (Figure 2) [25,26,27,28,29,30,31]. Thinned unripe fruit utilization opens new paths for orchard management and would increase the economic benefits for agricultural workers [32]. Although the usefulness of these bioactive compounds in unripe fruit was verified in many experiments, applications with high added value are still in the laboratory rather than in the factory. Most extraction methods and other applications of these high-added-value components have many disadvantages. For example, some bioactive components are susceptible to inactivation. On the other hand, the high cost of the extracting bioactive compounds from unripe fruit is an outstanding issue [33]. Therefore, the utilization and development of extraction methodologies for bioactive compounds and other applications of discarded unripe fruit require further study.

Thinned unripe fruits obtained from horticultural crops are rich in various active substances. However, traditional utilization was not employed by farmers, and this may detrimentally impact the biological and physico-chemical components of orchard soils. Therefore, the present review summarized the bioactive compounds contained in discarded unripe fruits, the beneficial health effects derived from the utilization of these bioactive substances, and methods for obtaining these compounds from thinned unripe fruits. After that, we roughly calculated the costs and benefits of obtaining bioactive compounds from discarded unripe fruits. This review has concluded and summarized the development and utilization of agricultural resources such as thinned unripe fruits from horticultural crops, and proposes issues for future research directions.

## 2. Bioactive Compounds from Thinned Unripe Fruits

### 2.1. Phenolic Compounds

During the growth and development of horticultural crops, the content of phenolic compounds in fruits decreases as fruit weight continues to increase because these compounds are mostly synthetized in fruit skins. Thereby, phenolic compound content in some unripe fruits is higher than in mature fruits [34], this has been confirmed in multiple reports, for example for grapes [35], apples [36], kiwifruits [37], and pomegranates [38]. Details are shown in Table 1.

During the growth and development of unripe fruits in certain fruit species, the content and profile of phenolic compounds are strongly affected by multiple factors. Labbé et al. [38] researched the differences in phenolic compound content in different ripening stages of pomegranate juices obtained from Wonderful, Chaca, and Codpa varieties. The author reported that the phenolic compound contents of pomegranate juices did not significantly differ between the different stages of ripening. However, significant differences were found between the different pomegranate varieties assessed. Interestingly, similar results were found in the study of strawberry. The difference in total phenolic content between the unripe and the ripe fruits was small, and the difference in total flavonoid content was significantly higher than in total phenolic content. The anthocyanin content of ripe fruits is significantly higher than that of unripe fruits, by up to 6 folds [39]. These details are shown in Table 1.

As mentioned above, phenolic compounds is a generic term that contains multiple hydroxyphenol compounds. Therefore, the composition of phenolic compounds is very complex, and their value does not always depend on total phenol content. Several researchers studied the phenolic composition of different unripe fruits in depth.

Bagheri et al. [41] detected seven phenolic compounds in unripe grape juices including rutin, gallic acid, hydroxybenzoic acid, syringic acid, *p*-coumaric acid, quercetin, and resveratrol. Redondo et al. [42] studied the content of monomeric phenols in six types of unripe stone fruits, namely apricots, cherries, peaches, peaches, plums, and nectarines. Monomeric phenols were rich in these by-products, and the main phenolic group analyzed included proanthocyanidins and quercetin 3-rutinoside. Salces et al. [43] and Sun et al. [44] detected multifarious phenolic compounds in unripe apples, Nakashima et al. [45] isolated 11 types of tetrameric procyanidins from unripe apples, and the detailed structural diversity was determined. Sun et al. [46] identified and individually quantified 102 individual polyphenols grouped in five major classes of phenolic compounds such as phenolic acids, phenol glycosides, flavanols and flavone alcohols, using ultra-performance liquid chromatography coupled with electrospray ionization triple quadrupole mass spectrometry (UPLC-Q TRAP-MS/MS) in thinned unripe pears. Other researchers detected a variety of monomeric phenolic compounds in unripe fruits such as pomegranate, kiwi, sweet orange peels, chinotto citrus and raspberry [19,37,40,47,48], as is shown in Table 2.

### 2.2. Non-Phenolic Bioactive Compounds

#### 2.2.1. Organic Acids

Unripe fruits contain a wide range of many other components with high bioactivity, including organic acids and polysaccharides. The acidity levels in unripe fruits are extremely high. Jančářová et al. [35] studied the change in acidity during the growth of different grape varieties. These authors reported that the titratable acid content of each grape variety increased (up to 40 g/L) initially, and the content of titratable acid decreased considerably as the fruit gradually ripened (up to 3.8 g/L). The content of titratable acid decreased rapidly as it matured until it became constant. In another report, de Matos et al. [49] produced “Verjuice” obtained from six unripe grapevine varieties at three different dates. These results reported that the “Verjuice” contained acidity levels that from 17.4 to 40.5 g/L. The malic acid and tartaric acid content in this juice varied from 10.9 to 30.4 g/L and from 5.5 to 14.0 g/L, respectively. The organic acid content in unripe peaches from Nagasawa hakuho and Mibaekdo varieties (*Prunis persica*) and Japanese apricots of the Backaha variety (*Prunus mume*) was also studied. These authors detected oxalic acid, tartaric acid, malic acid, and lactic acid in the three studied varieties. The contents of these organic acids in the fruits collected from the unripe peach varieties reached 612.8, 301.0, 495.5, and 314.7 mg/100 g of dry weight, respectively [50]. The main organic acids in strawberries are malic acid and citric acid, and the content of citric acid is generally higher than that of malic acid. With the increase in maturity, the content of organic acid gradually decreases [39].

#### 2.2.2. Polysaccharides

Apart from polyphenols, the polysaccharides obtained in unripe fruits also showed antioxidant and scavenging effects of free radicals, which may prevent and treat some chronic diseases [51,52]. To study the mechanical structure of plant cell walls, MacDougall et al. [53] used a gelation technique and found that pectin polysaccharides were the main component of cell walls. Based on this finding, the authors characterized tomato pectin polysaccharides and demonstrated that these compounds were primarily composed of rhamnogalacturonan. Galactose and arabinose are main polysaccharide units found in unripe apples, while mannose, rhamnose, glucuronic acid, galacturonic acid, glucose, and xylose contribute small amounts to its composition [54]. In addition, it was shown that the peels of unripe banana fruits contained rhamnose, trehalose, arabinose, xylose, mannose, galactose, glucose and uronic acid [55], as shown in Table 3.

Bananas are rich in starch, and starch content decreases significantly during maturity from 63.3 to 26.0 g/100 g [56]. Therefore, thinned unripe bananas have interesting potential for development as agricultural waste (Table 4). Espinosa-Solis et al. [57] studied the starches of unripe banana and mango fruits and compared the starch in these two unripe fruits to corn starch. The results showed that the content of amylose in unripe banana and mango fruits was higher than in corn. Moongngarm [58] showed that the powder extracted from banana fruits 105 days after banana flowering contained the most resistant starch, and it accounted for 48.9% of total starch. Menezes et al. [59] indicated that the content of resistant starch of unripe bananas reached 48.99 g/100 g—with the exception of mango meat and mango nuts. Patiño-Rodríguez et al. [60] showed that the starch content of unripe mango kernels was 48.79 g/100 g. Starch is also the main component in kiwi fruit when it is commercially picked. Furthermore, unripe kiwi fruits are also an interesting potential source of starch for utilization. The starch content of the kiwi variety Gold3 reached 40% 81 days after flowering and reached its highest starch content 137 days after flowering (58.45%) [61].

Polyphenols, organic acids, monosaccharides, starch, and other compounds are abundant in thinned unripe fruits, and each component plays a unique role. To reasonably use and develop the maximum extent of these agriculture wastes, it is necessary to deepen our research and explore its potential value. The present research on unripe fruit compounds lay a theoretical foundation for the development of extraction methodologies and understand the functional role of each component. 

## 3. “High Added Value” of Bioactive Components from Thinned Unripe Fruits

**Figure 3:**
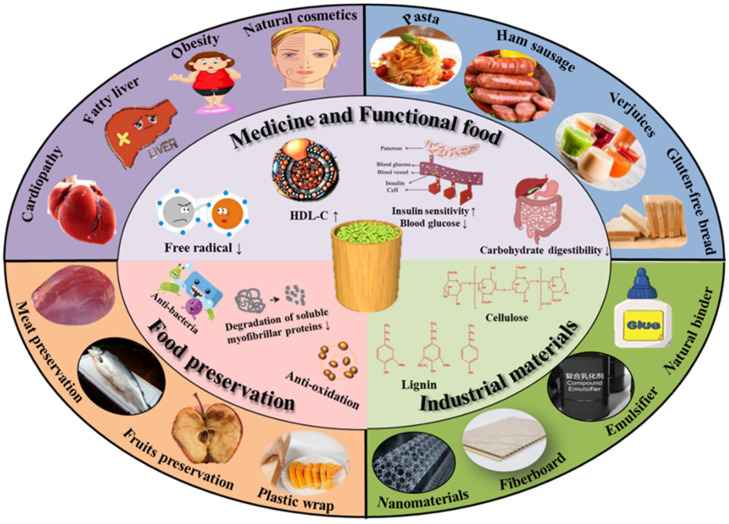
High added value of thinned unripe fruits (possible applications of unripe fruits in medicine, functional food, food preservation, and industrial materials).

Many studies reported that the inclusion of more fruits in the diet is good for our health because it may reduce the chance of many chronic diseases, such as cardiovascular disease, cancer, obesity and diabetes (Figure 3) [62,63]. These beneficial effects are attributed to the fact that fruits contain several phytochemical components, and phenolic compounds account for a large proportion [64,65,66,67,68,69]. The second apart of this review describes the bioactive components contained in unripe fruits, which occur at higher concentrations than mature fruits. These results are interesting because unripe fruits are good for human health, and the management of unripe fruits shows the positive aspects of the revalorization of byproducts in favor of a circular economy.

### 3.1. Utilization of Unripe Fruits for Crude Extract

An extract from unripe kiwi fruits was obtained by Abe et al. [70] and may play a beneficial role in diabetes through its ability to regulate the adipocytes’ differentiation and function; however, unripe *Musa paradisiaca* achieved the same effect because of its ability to adsorb blood sugar, and Bhinge et al. [71] verified this is greater for unripe than ripe and overripe fruit extracts. Some other studies showed that unripe apple pomace can be used as a functional food to achieve the same effect [72]. In addition, unripe grape juice consumption had a considerable effect on improving high-density lipoprotein (HDL-C) levels [31,73], and had important effects against food-borne pathogens such as *Bacillus cereus*, *Escherichia coli*, *Listeria monocytogenes*, *Salmonella Typhimurium*, and *Staphylococcus aureus*. Therefore, the juice from unripe grape contained antibacterial properties and could be considered a functional food as well [74]. Additionally, some researchers used unripe fruits for fresh fish feed, and the results showed a positive role of unripe fruits for feed [75]. However, beyond that, there is evidence that the extracts from unripe peaches and apples decreased the damage to the dermal–epidermal junction caused by UV-B radiation due to sun exposure [76,77].

Bioactive compounds from unripe fruits are variable, and if we only study the various effects of the unripe fruits as a whole, then many impurities may be obtained from the extraction reactions. Therefore, it is possible that the functional components of the unripe fruits cannot be used in a targeted manner. The commercialization of bioactive compounds may improve the utilization rate of unripe fruits by farmers. Consequently, the extraction of these components still needs further research. Some researchers simply extracted polyphenols, polysaccharides, organic acids, starches and other high-added-value components.

### 3.2. Utilizations of Unripe Fruits for Their Phenolic Composition

#### 3.2.1. Antioxidants

During plant growth and reproduction, polyphenols have a protective effect against pathogenic bacteria and have important antioxidant activities and their consumption is beneficial to human health [78,79]. These beneficial effects are primarily related to their anti-inflammatory activity [80], a lower incidence of a wide number of cancers and diabetes [81] and antithrombotic and heart protection activities [82,83]. Among all of the beneficial effects, antioxidant capacity is the most extensively studied. Phenolic compounds have benzene rings containing hydroxyl groups. Different polyphenol groups make diverse contributions to the antioxidant activity [84]. Phenolic composition plays a very important role in antioxidant capacity rather than phenolic content. Among them, proanthocyanidins B1, (−)-epicatechin and proanthocyanidins B2 had higher antioxidative activities than other polyphenols [85,86]. In other words, in terms of antioxidants, different polyphenolic compounds play different roles and the coordination between different polyphenols may determine the total antioxidant activity [87]. Therefore, it is still necessary to further study whether the main reasons for the high antioxidant activity of unripe fruits are total phenolic content, monomer phenolic composition, or the interaction between them.

Yue et al. [88] extracted phenolic compounds from unripe apple fruits and determined the antioxidant activity by evaluating 2,2-diphenyl-1-picrylhydrazyl (DPPH)-scavenging activity and the inhibition of lipid peroxidation. These authors indicated that the antioxidant activity against DPPH of the extracted polyphenols from unripe apples was higher than ripe fruits, and the polyphenols also effectively inhibited lipid peroxidation, with greater inhibitory activity than the lipophilic organic antioxidant compound butylated hydroxytoluene (BHT). Similarly, the antioxidant activity of polyphenol extracts from unripe stone fruits such as apricot, cherry, peach, plum, and nectarine, as well as from unripe grape fruits, was higher than from ripe fruits [42,89]. This suggests that many unripe fruits are potential antioxidants. 

#### 3.2.2. Natural Cosmetics 

With the continued research on polyphenols from unripe fruits, researchers are discovering other various study topics and different application aspects in addition to the antioxidant activity of unripe fruit polyphenols. Choi et al. [90] showed that polyphenol extracts of small-sized apples had a better inhibitory effect on tyrosinase than that of large apples. These results are interesting because thinned fruits are smaller and have a higher skin-to-fruit ratio than mature fruits, which controls the production of melanin. The authors elucidated that quercetin in the polyphenol extracts was closely related to the inhibition of tyrosinase. Unripe grapes can also counteract enzymatic browning, achieving the effect of natural skin whitening, cinnamic acid analogs including caffeic acid, coumaric acid and ferulic acid, suggesting that caffeic acid affects the tyrosinase inhibitory effect more than coumaric acid and ferulic acid. Early studies showed that polyphenolic compounds with hydroxyl or methoxy groups at the 4-position of cinnamic acid derivatives were potent inhibitors of tyrosinase [91]. In the same way, polyphenol extracts from peach juveniles also have the same utilization value [34,92]. Thus, the use of young fruit in cosmetic formulations should be a good choice. Sun et al. [44] found that porcine pancreas α-amylase (PPA) was inhibited by nine monomer phenols from unripe apples using in vitro essays, and tannic acid, chlorogenic acid and caffeic acid showed higher inhibition.

#### 3.2.3. Food Preservation 

It is precisely because polyphenols from unripe fruits have antioxidant activities and inhibitory effects on many enzymes that these biological properties give unripe fruit polyphenols a varied range of utilization. Sun et al. [93] studied the effect of polyphenols obtained from unripe apples on grass carp (*Ctenopharyngodon idellus*) surimi quality during cold storage at 4 °C. The research displayed that adding polyphenols from unripe apples to grass carp may have a dramatic effect on slowing down lipid oxidation and lead to a degradation of soluble myofibrillar proteins. Other interesting results from this report are that polyphenols from unripe apples also protected the functional characteristics of grass carp during the refrigeration process. They also increased the surface hydrophobicity and reduced their emulsification activity and stability. Among the studied phenolic compounds, chlorogenic acid played a major role in the aforementioned beneficial effects. 

#### 3.2.4. Biofilms

Owing to the antioxidant effect of polyphenols from unripe apples, a positive effect on the preservation of food and significant improvement in the antioxidant and bacteriostatic abilities of the chitosan film were observed. The author also tried to add polyphenols from unripe apples to edible packaging materials. The thickness, density, swelling, solubility, and opacity of the bioactive chitosan film were increased because of the addition of polyphenols extracted from unripe apples, but at the same time, there are some negative effects on the film, such as the water content, moisture permeability, and mechanical properties [94]. Nisar et al. [95] observed similar results when combining polyphenols from unripe apples with citrus pectin to make a biodegradable film. These authors suggested that polyphenols obtained from unripe apples had great potential in the production of thin biofilms. Similarly, unripe bananas and grapes also have antibacterial abilities. Chitosan–unripe banana peel films had an inhibitory effect on *Staphylococcus aureus* and *Escherichia coli*, which are generally present in wounds and showed the potential application for wound bacteriostasis [96]. Additionally, unripe grapes inhibited the fungal activity of Candida spp. and other dermatophytes [97].

#### 3.2.5. Functional Foods and Additives 

Unripe fruits are rich in polyphenols and good for human health, so their use as functional foods has become more popular in recent years. The transformation of polyphenols from unripe apples into functional foods is a very interesting topic currently, and it has promoted the development and utilization of discarded unripe fruits. Functional food such as industrially processed or natural food that when consumed regularly at efficacious levels has potentially positive effects on health beyond basic nutrition [98]. Verjuice and sour grape sauces produced from grape unripe fruits have important antioxidant activity and have extended the shelf life of foods. These characteristics result in antibacterial effects against different food-borne pathogens such as *Escherichia coli*, *Listeria monocytogenes*, Salmonella typhimurium and *Staphylococcus aureus* [99]. Therefore, unripe grape may be used as flavoring and acidifying agents, and may even be considered natural sanitizers. In light of food safety and human health, unripe grape products have the potential to replace chemical additives and preservatives, which may be related to the high total phenol and flavonoid contents [100]. However, the content of unripe grape used in the food industry is decided by various factors, such as processing conditions, variety, edaphoclimatic conditions of the growing area, and maturity at harvest, so there are still many unbroken difficulties in the industrialization of unripe grape products. 

The development of unripe fruits has a very positive effect on human health and the environment. Although polyphenols from unripe fruits may be used as functional foods, the taste of polyphenolic substances in unripe fruit is bitter, there are negative effects on the sensory quality of food. The addition of unripe fruits may also change the composition of polyphenolic substances. Bucalossi et al. [101] investigated how to make food products containing polyphenols from unripe fruits acceptable, in terms of sensory characteristics, to consumers and how to maximize the effect of polyphenols. An extract from thinned unripe grapes was used to fortify three plant-based food models: (i) beetroot purée (carbohydrates/acidic ph/sweet), (ii) pea purée (proteins/neutral ph/sweet) and (iii) potato purée (starch/neutral ph). In terms of both sensory quality and food functionality, beetroot purée was more suitable for offsetting the negative sensation induced by the polyphenols from unripe grapes.

### 3.3. Utilizations of Unripe Fruits Due to Non-Phenolic Bioactive Compounds

In unripe fruits, with the exception of polyphenols, there are many other bioactive compounds in unripe fruits with high value added, which also have antioxidant activity, free radical-scavenging activity, and many other useful functions.

#### 3.3.1. Health Profits 

Unripe banana fruits contain a large amount of starch, rich in carbohydrates that are not digested and absorbed by the human stomach and small intestine. Due to the presence of resistant starch, there is a low response in postprandial blood glucose. Long-term diet intervention on the body increases insulin sensitivity, and in vitro colonic fermentation produces more short-chain fatty acids. Unripe bananas have significant effects in improving diabetes, obesity and other diseases [102]. Sardá et al. [103] showed that healthy volunteers maintain blood sugar homeostasis, increased insulin sensitivity and satiety, and reduced hunger and daily energy intake when eating unripe banana powder three times per week for six weeks. Similarly, Rosado et al. [104] showed that the consumption of resistant starch from unripe bananas in mice allowed lower gains in body mass than mice consuming non-supplemented starch. The accumulation of liver fat was less in mice receiving unripe bananas than the control non-supplemented mice and the liver was protected by the reduced production of short-chain fatty acids due to the addition of at least 15% unripe bananas in the diet. 

Many researchers characterized the polysaccharides of unripe apples and some research examined its development in recent years due to its potential functional value. Dou et al. [54] characterized the polysaccharides of unripe apples, and polysaccharides extracted from unripe fruits possessed antioxidant capacity. Polysaccharides were obtained from unripe apples by Chen et al. [105] and shown to possess the ability successfully reduce obesity-related liver metabolic disorders via activation of the respiratory function of liver mitochondria in mice.

#### 3.3.2. Functional Foods and Additives 

Unripe fruits contain important organic acids, and it is possible to produce unripe fruit juices or powders as natural acidifier condiments [49]. Some starchy unripe fruits, such as banana, mango, and kiwi can be used as a substitute for flour or starch to make many functional foods. Castaño et al. [106] used unripe banana flour rather than wheat to produce gluten-free bread. Compared with wheat bread, gluten-free bread with 8% and 15% of banana flour resulted in higher fiber content and lower carbohydrate content, and the same results were found in Patiño-Rodríguez’s [107] study. However, gluten-free products have a slightly different texture compared to gluten products, and taste will be defective. Incidentally, Aguirre [108] reported that the addition of hydrocolloids and pregelatinized starches such as hydroxypropyl methylcellulose and pregelatinized unripe banana flour improved the taste of gluten-free products. Moreover, other researchers replaced fat with unripe bananas to make ham sausages [109], pound cakes [110] and other foods, which reduced the use of fat and sugar. The revalorization of unripe bananas has allowed us to obtain healthier foods without affecting sensory characteristics. 

#### 3.3.3. Ethanol Production 

Waghmare et al. [111] made a powder from unripe banana skin, and produced a 49.2% *w*/*w* reduction in sugars by acid hydrolysis, and *Saccharomyces cerevisiae* was screened for ethanol conversion efficiency. On the basis of optimizing the conditions of fermentation, 35.5 g/L ethanol was produced. In this condition, unripe banana peels were also a potential resource for ethanol production. 

Global warming is one of the greatest issues in the wine industry because wines have an increasingly high alcoholic content due to the high accumulation of soluble solids in berries. Certain studies showed that unripe grapes could be used to produce low-alcohol wines or reduce the alcohol content of the wine by mixing unripe grapes with grapes that had reached harvest maturity [112,113]. “Double harvest” is the technique that decreases the alcoholic content in wines and improves the acidity in a natural form. This technique also takes advantage of the grapes removed by cluster thinning [114]. In addition to reducing alcohol content, Fia et al. [115] also found that the addition of unripe grape fruit extracts to wine could play a role in protecting wine color during aging, and could be an interesting substitute for SO_2_ during wine ageing. Considering that unripe fruits lack phenolic maturity, and bring an excess of astringency and herbaceous aromas, Junior et al. [116] found that *S. bacillaris* can be used to increase glycerol content and malic acid degradation, thereby balancing the lack of palate fullness caused by low alcohol content and masking ‘vegetative’ odors. 

#### 3.3.4. Industrial Materials 

Sucrose and glucose are the main sugars in unripe peach fruits, and citric acid is the main organic acid. Compared with mature fruits, unripe fruits have higher contents of organic acids and a lower sugar content than ripe fruits, which may be helpful to the formation of carbon materials [117]. For this reason, unripe fruit may be a good source of nanomaterials. For example, photoluminescent carbon dots (CDs) are a new class of nanoparticles with exceptional advantages, such as low toxicity, high chemical stability, good biocompatibility, excellent optical performance, and low photo bleaching, and have therefore received wide interest. At the same time, nitrogen-doped carbon dots (N-CDs) are also receiving interest due to highly enhanced photoluminescence [25,118].

After unripe bananas were made into banana powder, Martínez et al. [119] produced a natural polymer based on 50% thermoplastic unripe banana flour (TPF) mixed with metallocene-catalyzed polyethylene (mPE), which was a robust, elastic, and non-brittle material. With these characteristics, the blends were ideal for the design of biodegradable plastics. The shells of unripe coconuts are very rigid and contain a large amount of cellulose and lignin. Lignin from coconut husks may be used as a natural binder with high temperature and pressure. Based on these findings, a convenient and favorable technology was developed from coconut husks to produce high-strength–high-density panels without the addition of chemical binders. Junior et al. [120] reported that coir-based fiberboards may be applied to furniture, wallboards, floors, and coatings without the need to cut down trees. Unripe coconut husk as a raw material with potential for use in fiberboards without binders was examined. The results showed that coconut shell material played a crucial role in the production of fiberboards. Additionally, there were other studies also showing that polysaccharides from unripe apples had emulsification properties [32].

Based on the published research, the utilization of unripe fruits involves different fields such as medicine, food preservation, functional foods, industrial materials, and environmental care. Therefore, the use of unripe fruits at this stage is extremely important. Several wastes are produced during horticultural production and we must identify an economical method to recycle agricultural waste. The collection of bioactive components from unripe fruits is of wide importance, and one critical step is to maximize the value of their extraction using different methodologies, which is discussed in the following sections.

## 4. Methods to Extract Bioactive Components from Thinned Unripe Fruits

### 4.1. Methods to Extract Phenolic Compounds

**Figure 4:**
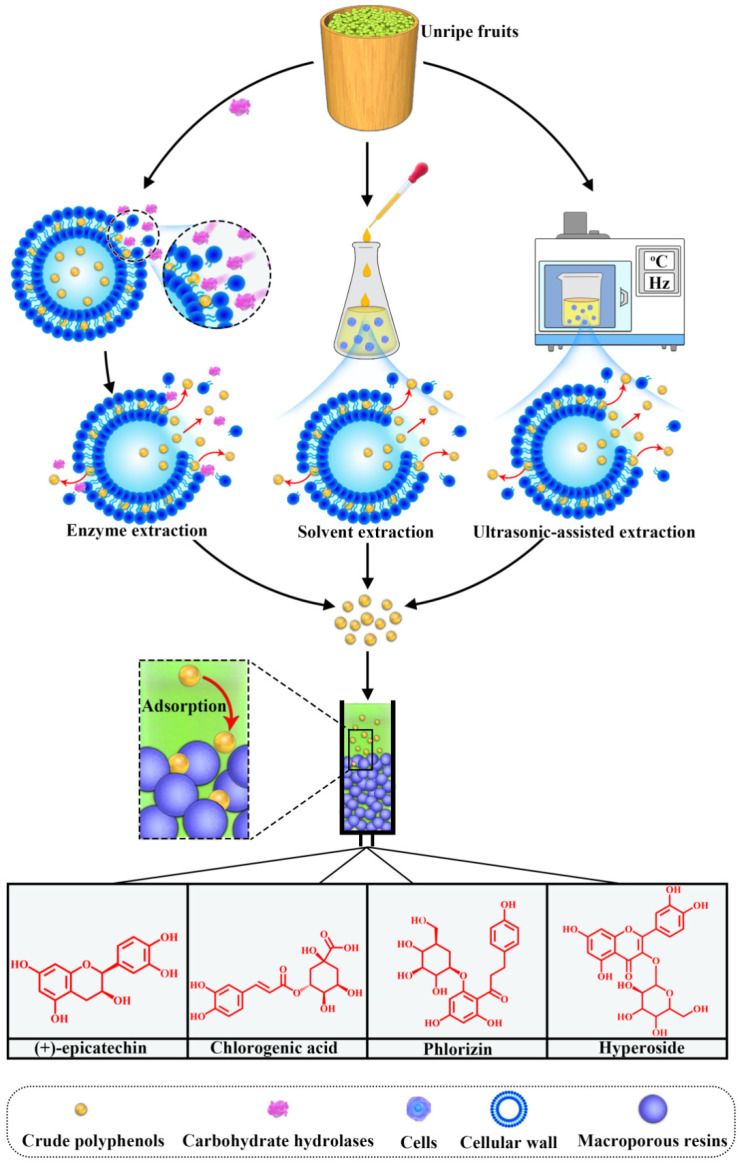
The methods of extracting polyphenol.

It has been described above that unripe fruits are one source of polyphenolic compounds. According to many experiments, because of the important effects on human health and food preservation, polyphenols of unripe fruit may have the potential to replace traditional chemical inhibitors and preservatives. The development of polyphenols from unripe fruits would greatly impact environmental resources. Present extraction methods of phenolic compounds include solvent extraction [121], subcritical water extraction [122], microwave-assisted extraction [123] and enzyme extraction methods [124], but there is no large-scale extraction technology to extract polyphenols from unripe fruits. Therefore, the economic costs of this extraction are high. In addition, the instability of the polyphenols, due to their presence in bounded or polymerized forms, in fruits limits their extraction, purification and equipment technology [15]. In recent years, many scholars performed a series of studies on the role of polyphenols from unripe fruits and the pretreatment of unripe fruits, including solvent extraction, microwave-assisted extraction and enzyme extraction method are described as follow (Figure 4), which has laid a certain foundation for the extraction of polyphenols.

To improve the extraction efficiency of plant polyphenols, years ago, researchers introduced carbohydrate hydrolases (such as pectinase, cellulase, hemicellulose, and glucanase) to release complex polyphenols from cell walls (Figure 4) [125,126,127]. Zheng et al. [128] showed how to increase the extraction of phenolic compounds from unripe apple fruits by selecting a carbohydrate hydrolase (Viscozyme^®^ L). Subsequently, Zheng [124] enhanced the optimal conditions for the extraction of phenolic compounds from unripe apples, and the extraction rate of polyphenols was improved compared to Zheng et al. [128]. The total phenol and caffeic acid contents were approximately 2- and 13-fold higher than that of the control, respectively.

Shojaee-Aliabadi et al. [129] extracted polyphenols from unripe grapes using a solvent extraction method and an ethanol solution as the extractant agent and discussed the impact conditions such as extraction time, extraction temperature, and ethanol concentration, optimized by the response surface methodology. The results elucidated that the amounts of extracted polyphenols and the elimination of DPPH were significantly affected by the time, temperature, and ethanol concentration of the extraction. After conditional optimization, phenol extraction was improved. The extracted content of polyphenols was 0.39 g GAE/100 g, and the amount of DPPH elimination was 91.01%. Proanthocyanidins from unripe sapodilla (*Manilkara zapota*) fruits were also isolated using solvent extraction through gel filtration media (Sephadex LH-20) [130]. Yue et al. [88] obtained polyphenolic compounds from unripe apple fruits by ultrasonic-assisted extraction and studied the effects of ultrasonic power, extraction time, temperature, and ethanol concentration on total polyphenol yield by the response surface method. The results indicated that the total polyphenol yield was 13.26 ± 0.56 mg GAE/g. Several experiments showed that ultrasound-assisted extraction significantly improved the extraction rate of phenolic compounds, with important reductions in reaction times compared to solvent extract methodologies [41]. In recent years, Fia et al. [131] obtained bioactive compounds from unripe grape through a green extraction. The crushed unripe grapes were extracted by the maceration system, the use of dry ice and membrane pneumatic press to prevent oxidative damage and increase the extraction yield.

In terms of the purity of extracted polyphenols, some studies elucidated that the use of macroporous resins is generally recommended because they possess suitable adsorption and desorption capabilities for polyphenol extraction [132]. The X-5 macroporous resin was selected for the purification of polyphenols extracted from unripe apple fruits. The phenolic content increased from 35.17% to 74.64%, with a recovery yield of 89.35%, which is a 2.12-fold increase. These authors selectively purified chlorogenic acid and phlorizin using X-5 and polyamide resins. The contents of chlorogenic acid and phlorizin were 15.20% and 97.52%, with recovery yields of 89.16% and 64.95%, respectively [133]. Sun et al. [134] performed dynamic adsorption and desorption by X-5, polyamide resins, and a strippant of ethanol solution. Chlorogenic acid, epicatechin, hyperoside, and phlorizin were obtained from unripe apples with purities of 96.21%, 95.34%, 95.36%, and 97.36%, respectively.

### 4.2. Methods to Extract Non-Phenolic Bioactive Compounds

In the development of unripe fruits, few studies were performed on the extraction and purification of non-phenolic bioactive compounds. Due to the rich fiber content in unripe banana fruits, fiber powder from unripe banana was obtained by Rodriguez-Ambriz et al. [135] via a series of acidolyzed, liquefied, gelatinized, enzymatic hydrolyzed steps. In addition to fiber, the unripe banana is rich in starch. Khoozani et al. [136] studied the effect of different drying conditions, such as oven air-drying and freeze-drying, on the starch content, thermal properties and other physico-chemical parameters of unripe bananas flour. Banana flour dried using oven air at 50 °C and freeze-drying presented a higher resistant starch content. The first method showed the highest amylose content and degree of crystallinity and the lowest gelatinization temperature among the green banana flour samples (4.69 °C). These authors concluded that drying the unripe banana at 50 °C resulted in fewer negative effects on resistant starch content than those of the other drying temperatures studied and the flour produced exhibited similar physico-chemical characteristics compared to freeze-dried flour.

**Figure 5:**
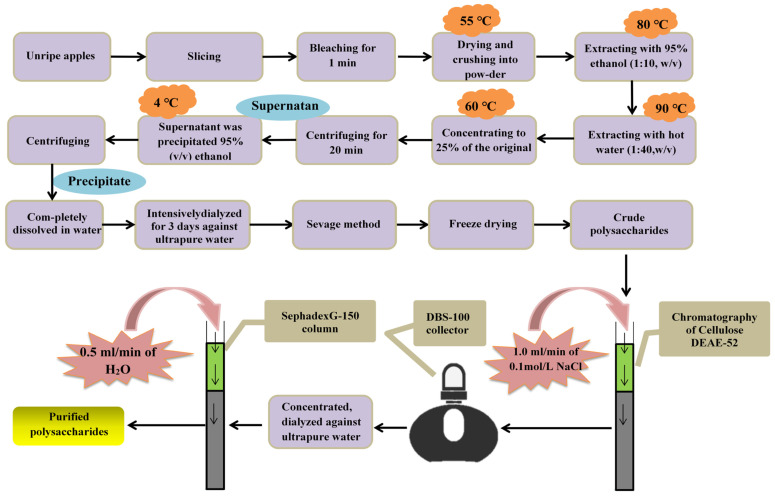
The entire process of the separation and purification of polysaccharides.

As mentioned above, the polysaccharides in unripe fruits have beneficial effects on the human body for example as antioxidant, so the extraction of polysaccharides is also worthy of further study (Figure 5). Dou et al. [137] used hot water extraction in a 70% ethanol solution to remove polyphenols and the Sevage method to remove proteins and isolate crude polysaccharides from thinned unripe apples. Crude polysaccharides were separated using DEAE-52 cellulose column chromatography and eluted sequentially at a gradient of 0–0.5 moL/L NaCl solution, with a flow rate of 1.0 mL/min. Samples were obtained using a DBS-100 collector. The total sugar mass fraction of crude polysaccharides after isolation and purification was 99.24% without impurities such as nucleic acids, proteins and polyphenols. Polysaccharides were also obtained from unripe apple fruits via hot water extraction at 88 °C for 122 min [41]. After multiple elution using macroporous resin, the content of extracted polysaccharide reached 92%, and they did not contain impurities such as proteins and polyphenols.

## 5. Costs and Benefits 

As mentioned in the previous sections, thinned unripe fruits are generally discarded in the orchard, which generates much waste. Some researchers successively characterized the bioactive components of unripe fruits. These studies were performed to extract high-added-value components from unripe fruits for use in different fields such as in the cosmetic, pharmaceutical, food, and plastic industries or in energy production. The development of a fledgling industry of unripe fruit management could generate significant economic benefits in addition to the ecological benefits involved in the utilization of agricultural waste. Therefore, the discussion of costs and benefits is an inevitable topic in the process of unripe fruit management.

We will now take into account the report published by Sun et al. [134] that it is possible to roughly calculate the costs and benefits of the development of unripe apple fruit management. Sun et al. [133] used solvent extraction technology to extract polyphenols from 100 g fresh unripe apple fruit, and used resins to separate and purify crude polyphenols. The experimental consumables for the extraction of bioactive compounds from unripe fruits included hydrochloric acid, sodium hydroxide, ethanol, ethyl acetate, sodium sulfite, macroporous adsorption resin, chromatography columns, and peristaltic pumps. Unripe fruits are agricultural waste with extremely low commercial value and potential use, so we do not take a cost for them into account. The total cost was $111.198, as shown in Table 5. 

**Figure 6:**
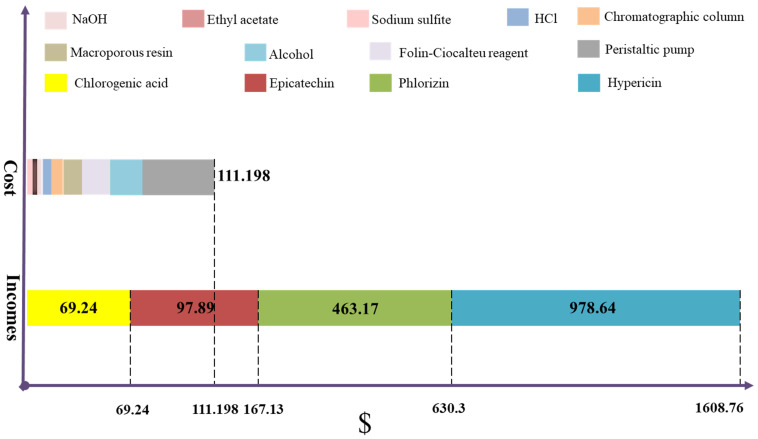
Costs and incomes produced from the extraction of monomeric phenols from thinned fruits.

In the course of the experiment, 0.534 g chlorogenic acid, 0.382 g epicatechin, 39.54 mg hypericin, and 0.512 g phlorizin were obtained from 100 g fresh unripe apple fruit, and all with a purity higher than 95%. We calculated the outputs according to the lowest price of the corresponding reagent published by the official website of the Sigma-Aldrich company. The total income produced from unripe fruit waste management was $1608.76, as shown in Table 6. The economic benefits are close to 14-fold more than the costs, as shown in Figure 6. However, if the development of the management of unripe fruits waste is extended to an industrial scale, the costs related to the use of water, electricity, man-hour labor costs, sewage treatment, raw material costs and storage should be considered. Nevertheless, the development of industrialization will multiply the number of bioactive compounds extracted and the number of monomeric phenols and many other by-products obtained, such as sour agents and polysaccharides. It is obvious that the benefits of unripe fruit development are very impressive. 

In addition, unripe fruits may also be used as food additives to replace the original materials to develop functional foods, such as unripe banana flour instead of wheat flour to make bread slices [138], and cakes that are rich in high dietary fiber [139]. Moreover, unripe banana powder may also be used as a snack instead of tapioca starch [140]. De Matos et al. [141] mentioned that unripe oranges could be used in salad dressings due to their low pH and antioxidant activity, and Öncül et al. [142] added unripe grape fruits to fruit juices and jams. There were no other extra costs added, and natural antioxidants from unripe fruits may increase the stunting of product to achieve the aim of increasing revenue. This use will promote the development of a circular economy that can reduce the environmental impacts of the non-management of thinned unripe fruits and improve the economic benefits of farmers. 

## 6. Outlook

In summary, the thinned unripe fruits from horticultural crops are indeed a potential agricultural resource. Thinned fruit bioactive components, such as polyphenols, polysaccharides and organic acids, may be used in the food preservation, food additives, medicine, materials, cosmetics and other industries. However, the development of unripe fruits faces many challenges simultaneously.

Since unripe fruits are still growing, their bioactive components undergo different changes before thinning. The monitoring and recording of some physico-chemical parameters may be performed to establish a database of the bioactive components at different ripening stages of horticultural fruits. This measurement would help orchard coordination choose a suitable thinning date to maximize the content of bioactive compounds from thinned fruits without affecting the potential productivity of the orchard. Most of the collected thinned fruits are fallen fruits in soils, which presents serious problems in quality such as rotting, dehydration and the presence of insects. With the frequent use of inorganic fertilizers and pesticides, heavy metal and pesticide pollution of unripe fruits is inevitable. Therefore, collection, storage, preservation, and grading after thinning are equally important. Toxicity detection is also an essential link before unripe fruit are used as raw materials for semi-industrial and industrial purposes. How to reduce or eliminate potential harm from unripe fruit to health is one of the main challenges we must face in the future. 

As mentioned above, the blend fermentation of unripe grapes and wine grapes can solve the disadvantage of “high sugar and low acid” caused by global warming. However, the addition of unripe fruit in wines may bring unpleasant green tastes to the wines. The removal of green tastes is a problem that needs to be solved in this process. In addition, different wine-making materials have different sugar and acid ratios, so the optimal amount of unripe fruit is often adjusted according to the quality of wine grapes.

Among the unripe fruits of many horticultural crops, unripe apples are the most investigated. Based on these studies, phenolic compounds are the most abundant bioactive components of unripe fruits. Although the polyphenol content in unripe fruits is higher than mature fruits, the proportion of total phenols extracted from unripe fruits is small. In addition, many resources are wasted in the extraction of monomeric phenols from unripe fruits. Therefore, future development and utilization of unripe fruits resources may resort to the “biorefinery” concept to fully extract and use high-value-added by-products from thinned fruits. Arevalo-Gallegos et al. [143] defines biorefinery as a sustainable method to convert biological raw materials (such as biomass) into energy in an economical and environmentally friendly manner. Thinned fruits as raw materials for biomass achieve faster material transfer due to their organoleptic wealth and internal morphological structure. Compared to wood or herbal biomass, acids, alkali catalysts, enzymes and microorganisms have higher activity in unripe fruits, which reduces the processing time. After the proper extraction of valuable bioactive compounds such as polyphenols, organic acids, polysaccharides, and starch from unripe fruits, heat may be used to quickly obtain biogas, bio-oil, and biochar via pyrolysis. These mixtures may be used to obtain sufficient energy [20,24].

## 7. Conclusions

This review summarized the comprehensive utilization of thinned unripe fruits from horticultural crops, especially apples, grapes, bananas, peaches, mangos, and kiwis. Thinned unripe fruits are often abandoned in orchards and cannot be optimally utilized. These unripe fruits are rich in several bioactive compounds, such as polyphenols, polysaccharides and organic acids. These compounds prevent oxidation, inhibit bacteria, reduce blood sugar, improve obesity, and possess emulsifying, thermoplastic, and stability properties. Increasingly, researchers have begun to extract high-value-added components from unripe fruits to generate a wide range of by-products for use in many industries such as medicine, food preservation, and polymer materials. Therefore, unripe fruits have great potential in the revalorization of agricultural wastes, which could improve economic benefits for farmers. Solving the environmental pressure of unripe fruits may also create greater economic benefits for agricultural workers.

## Figures and Tables

**Table 1:** Comparison of total phenol content in unripe and mature fruits.

Unripe Fruits	Cultivars	Maturity Stages	Total Phenol Content		Reference
			Unripe	Ripe	
Grape	Müller-Thurgau, Pinot Blanc, Sauvignon, Muscat Ottonel	40 days after flowers	5000 mg/L	104 mg/L	[35]
Apple	*Fuji*	25 days after full bloom day	360 mg/100 g	40 mg/100 g	[36]
Kiwifruit	Zesy002, Zesy004, Hayward	20 days after fruit set	71.67 mg/100 g	9.05 mg/100 g	[37]
Pomegranate	Wonderful	——	1300 mg /L	1910 mg/L	[38]
	*Chaca*		1850 mg/L	2130 mg/L	
	*Codpa*		1700 mg/L	1610 mg/L	
Sweet Orange Peels	Navel variety	physiologically matured with green peel	5.27 mg/100 g	9.40 mg/100 g	[40]

——: not mentioned in the literature.

**Table 2:** Phenolic compounds detected in unripe fruits.

Unripe Fruits	Cultivars	Maturity Stages	Mono-Phenol Component						Reference
			Phenolic Acids	Flavanols	Flavanones	Flavonols	Stilbenes	Others	
Apricots	*Pink Cot*	42 and 48 days after full bloom	Neochlorogenic acid, chlorogenic acid, 4-*p*-coumaroylquinic acid, 4-caffeoylquinic acid, 3-feruloylquinic acid, and 3-*p*-coumaroylquinic acid	Proanthocyanidins	Quercetin-3-*O*-rutinoside and kaempherol-3-*O*-rutinoside	——	——	——	[42]
Cherries	*13S-20-09*	42 and 48 days after full bloom	Neochlorogenic acid, chlorogenic acid, isochlorogenic acid, 4-*p*-coumaroylquinic acid, 4-caffeoylquinic acid, 3-feruloylquinic acid, and 3-*p*-coumaroylquinic acid	Proanthocyanidins	Quercetin-3-*O*-rutinoside and kaempherol-3-*O*-rutinoside	——	——	——	
Peaches	*UFO-3*, *Royal Glory*, *Laura*	42 and 48 days after full bloom	Neochlorogenic acid, chlorogenic acid, isochlorogenic acid, 4-*p*-coumaroylquinic acid, 4-caffeoylquinic acid, 3-feruloylquinic acid, and 3-*p*-coumaroylquinic acid	Proanthocyanidins	Quercetin-3-*O*-rutinoside, quercetin-3-*O*-hexoside, kaempherol-3-*O*-hexoside, and kaempherol-3-*O*-rutinoside	——	——	——	
Plums	*Tolosa*	42 and 48 days after full bloom	Neochlorogenic acid, chlorogenic acid, Isochlorogenic acid, 4-*p*-coumaroylquinic acid, 4-caffeoylquinic acid, 3-feruloylquinic acid, and 3-*p*-coumaroylquinic acid	Proanthocyanidins	Quercetin-3-*O*-rutinoside	——	——	——	
Grape	*Thompson Seedless*	——	Ggallic acid, benzoic acid, hydroxybenzoic acid, syringic acid, and *p*-coumaric acid	——	Quercetin and rutin	——	Resveratrol	——	[41]
Apple	*Fuji*, *Nagafu 2*	30 days after blossoming	5-Caffeoylquinic acid, 4-*p*-coumarylquinic acid, chlorogenic acid, caffeic acid, and phenylalanine ester	(+)-Catechin, (−)-epicatechin, and procyanidin B2	Rutin, quercetin quercetin-3-*O*-galactoside, quercetin-3-*O*-rhamnoside, isoquercetin, and quercetin	Phloretin-2′-*O*-xyloglucoside, hydroxyphospholipid monoglycoside, hydroxychrometin diglycoside, and hyperoside	——	——	[44]
Pomegranate	*Borde de Albatera 1*, *PiñónTierno deOjós PiñónTierno deOjós5*, *Mollar de Elche* 14 et al. (all of 9)	Middle of June to the first week of July (Orihuela, Alicante, Spain)	——	Ellagic tannin, gallic acyl-hexahydroxy, dibenzoyl-hexanoside, and gallic acyl-glucoside	——	——	——	——	[19]
Kiwifruit	*Zesy002*, *Zesy004*, *Hayward*	20 days after fruit set	Gallic acid, protocatechuic acid, chlorogenic acid, caffeic acid, vanillic acid, *p*-Coumaric acid, and ferulic acid	Catechin epigallocatechin gallate, and epicatechin	Rutin, quercitrin, and quercetin hyperoside	——	——	——	[37]
Pears	*Fengshui*, *Cuiguan*, *Dangshansuli* et al. (all of 10)	20 days after full bloom	Quinic acid, cinnamic acid isomer, chlorogenic acid, 1-*O*-caffeoylquinic acid, 4-*p*-coumaroylquinic acid, caffeoylshikimic acid, 3-*O*-feruloylquinic acid, caffeoyl-malonyl-methylcitric acid, isochlorogenic acid A, and *p*-coumaroylcaffeoylquinic acid	(+) -Catechin, (−)-epicatechin, and procyanidin dimer	——	——	——	Arbutin, caffeoylarbutin, *p*-coumaroylarbutin, and *p*-coumaroylarbutin	[46]
Orange	*Navel variety*	Physiologically matured with green peel	Caffeic acid	Catechin and epicatechin	Rutin, quercitrin, quercetin, naringin, kaempferol, and luteolin	——	——	——	[40]
Raspberry	*Rubus chingii Hu*	Green fruit stage	Ellagic acid	(+)-Catechin, (−)-epicatechin, and proanthocyanidin B1	Rutin, quercetin, kaempferol, quercetin-3-*O*-glucuronide, kaempferol-3-*O*-rutinoside, and kaempferol-3-*O*-glucoside	——	——	——	[48]

——: not mentioned in the literature.

**Table 3:** Organic acids and monosaccharides in unripe fruits.

Active Components	Unripe Fruits	Cultivars	Maturity Stages	Component Characterization	Reference
Organic acids	Grape	Glera, Chardonnay, Sauvignon Blanc, Merlot, Cabernet Franc and Cabernet sauvignon	Bunch closure	Tartaric acid and malic acid	[49]
	Peach	Mibaekdo and Nagasawa Hakuho	30–35 days after flowers	Oxalic acid, tartaric acid, malic acid, and lactic acid	[50]
	Strawberry	Seolhyang, Janghee, and Maehyang	50% of fruit surface turns into red color	Malic acid and citric acid	[39]
Monosaccharide	Grape	Glera, Chardonnay, Sauvignon Blanc, Merlot, Cabernet Franc and Cabernet Sauvignon	Bunch closure	Glucose and fructose	[49]
	Tomato	——	——	Rhamnogalacturonan	[53]
	Apple	——	30 days after blossom	Galactose, arabinose, mannose, rhamnose, glucuronic acid, galacturonic acid, glucose, and xylose	[54]
	Banana	*Musa sapientum*	——	Rhamnose, trehalose, arabinose, xylose, mannose, galactose, glucose, and uronic acid	[55]
	Strawberry	Seolhyang, Janghee, and Maehyang	50% of fruit surface turns into red color	Fructose, glucose, and sucrose	[39]

——: not mentioned in the literature.

**Table 4:** Starch content in unripe fruits.

Unripe Fruits	Cultivars	Maturity Stages	Apparent Amylose Content [%]	M_w_ × 10^8^ [g/mol]	R_z_ [nm]	Reference
Bananas	Macho	——	36.2	3.371 ± 0.179	267.10 ± 5.515	[57]
Mango meat	Tommy Atkins	——	31.1	5.013 ± 0.177	297.85 ± 1.202	[58]
Mango nuts	——	——	23.0	——	——	
Kiwifruit	Hayward	45 days after flowers	10.0	——	——	[61]
	Gold3	53 days after flowers	15.4	——	——	

——: not mentioned in the literature.

**Table 5:** Costs produced from the extraction of monomeric phenols from thinned fruits.

	Consumable	Material Usage	Market Price ($)	Total Price ($)
Consumable test	HCl	200 mL	2.28/500 mL	0.912
	NaOH	200 g	1.57/500 g	0.628
	Alcohol	2000 mL	2.71/500 mL	10.84
	Ethyl acetate	800 mL	1.99/500 mL	3.184
	Sodium sulfite	300 g	3.14/500 g	1.884
	Folin–Ciocalteu reagent	10 mL	10.57/50 mL	2.11
	Macroporous resin	2000 g	7.8/1000 g	14.6
	Column	2	2.85/set	5.7
	Peristaltic pump	1	71.34/set	71.34
Total amount: $111.198				

**Table 6:** Incomes related to the obtaining of monomeric phenols from thinned fruits.

	Product	Yield/100 g	Market Price ($)	Total Price ($)
High-added-value components	Chlorogenic acid	0.534 g	129.66/g	69.24
	Epicatechin	0.382 g	256.25/g	97.89
	Hypericin	39.54 mg	495.01/20 mg	978.64
	Phlorizin	0.512 g	904.63/g	463.17
Total amount: $1608.76				

## Data Availability

Not applicable.
